# Hyperactivation of proprioceptors induces microglia-mediated long-lasting pain in a rat model of chronic fatigue syndrome

**DOI:** 10.1186/s12974-019-1456-x

**Published:** 2019-03-30

**Authors:** Masaya Yasui, Yuki Menjyo, Kyohei Tokizane, Akiko Shiozawa, Makoto Tsuda, Kazuhide Inoue, Hiroshi Kiyama

**Affiliations:** 1 Department of Functional Anatomy and Neuroscience, Nagoya University Graduate School of Medicine, 65 Tsurumaicho, Showa-ku, Nagoya, Aichi 466-8550 Japan; 2 Department of Anatomy, Aichi Medical University, 1-1 Yazakokarimata, Nagakute, Aichi 480-1195 Japan; 3 Department of Molecular and System Pharmacology, Graduate School of Pharmaceutical Sciences,, Kyushu University, 3-1-1 Maidashi, Higashi-ku, Fukuoka, Fukuoka 812-8582 Japan

**Keywords:** Microglia, Pain, Chronic stress, Chronic fatigue syndrome, Fibromyalgia, Proprioceptor

## Abstract

**Background:**

Patients diagnosed with chronic fatigue syndrome (CFS) or fibromyalgia experience chronic pain. Concomitantly, the rat model of CFS exhibits microglial activation in the lumbar spinal cord and pain behavior without peripheral tissue damage and/or inflammation. The present study addressed the mechanism underlying the association between pain and chronic stress using this rat model.

**Methods:**

Chronic or continuous stress-loading (CS) model rats, housed in a cage with a thin level of water (1.5 cm in depth), were used. The von Frey test and pressure pain test were employed to measure pain behavior. The neuronal and microglial activations were immunohistochemically demonstrated with antibodies against ATF3 and Iba1. Electromyography was used to evaluate muscle activity.

**Results:**

The expression of ATF3, a marker of neuronal hyperactivity or injury, was first observed in the lumbar dorsal root ganglion (DRG) neurons 2 days after CS initiation. More than 50% of ATF3-positive neurons simultaneously expressed the proprioceptor markers TrkC or VGluT1, whereas the co-expression rates for TrkA, TrkB, IB4, and CGRP were lower than 20%. Retrograde labeling using fluorogold showed that ATF3-positive proprioceptive DRG neurons mainly projected to the soleus. Substantial microglial accumulation was observed in the medial part of the dorsal horn on the fifth CS day. Microglial accumulation was observed around a subset of motor neurons in the dorsal part of the ventral horn on the sixth CS day. The motor neurons surrounded by microglia were ATF3-positive and mainly projected to the soleus. Electromyographic activity in the soleus was two to three times higher in the CS group than in the control group. These results suggest that chronic proprioceptor activation induces the sequential activation of neurons along the spinal reflex arc, and the neuronal activation further activates microglia along the arc. Proprioceptor suppression by ankle joint immobilization significantly suppressed the accumulation of microglia in the spinal cord, as well as the pain behavior.

**Conclusion:**

Our results indicate that proprioceptor-induced microglial activation may be a key player in the initiation and maintenance of abnormal pain in patients with CFS.

**Electronic supplementary material:**

The online version of this article (10.1186/s12974-019-1456-x) contains supplementary material, which is available to authorized users.

## Background

Functional somatic syndrome (FSS) is characterized by the presence of multiple cryptogenic symptoms such as severe fatigue, pain, sleep disturbance, malaise, and cognitive dysfunction [1–3]. FSS includes disorders such as chronic fatigue syndrome (CFS), fibromyalgia syndrome (FMS), and irritable bowel syndrome (IBS). Although these diseases exhibit substantial overlap with regard to symptoms, their etiologies remain largely unclear [4]. Previous research studies have indicated that the pathogenesis of such disorders may involve the disintegration of the nervous, immune, and endocrine systems due to prolonged psychological and physiological stress [5, 6]. Animal models may therefore help to elucidate the molecular and cellular mechanisms underlying the pathogenesis of these disorders.

Several recent studies have utilized a chronic or continuous stress-loading (CS) rat model that partially mimics the symptoms associated with CFS and FMS [6–11]. In this model, a rat is placed in a cage with a very low level of water (1.5 cm in depth) for 1–5 days to induce a continuous level of stress (i.e., CS). Using this model, researchers have demonstrated that CS induces gene expression in several organs, as well as dramatic changes in the pituitary gland at the molecular and cellular levels [7–9, 11–13]. Rats exposed to CS exhibit significant activation of melanotrophs in the intermediate lobe and suppression of somatotrophs in the anterior lobe, as evidenced by the active secretion of alpha-melanocyte stimulating hormone (α-MSH) and significant decreases in growth hormone levels *in sera* [9, 10]. Notably, recent evidence suggests that patients with CFS exhibit higher levels of α-MSH in the blood, and thus, the rat CS model may be useful for further investigations of CFS [14]. Interestingly, alterations in pituitary hormone levels are caused by changes in dopaminergic and growth hormone-releasing hormone (GHRH) neurons in the hypothalamus, suggesting that CS-induced impairments of the endocrine system are due to changes in central nervous system (CNS) neurons.

Another characteristic symptom of CFS and FMS is abnormal muscle pain (e.g., hyperalgesia) [1–3]. We previously demonstrated that rats under CS exhibited mechanical allodynia at the plantar surface and mechanical hyperalgesia at the anterior tibialis (i.e., muscle pain) [6]. Although no signs of inflammation or injury were observed, the rats exhibited microglial accumulation and activation in the lumbar dorsal horn (L4–6). Minocycline, an inhibitor of microglia activation, significantly attenuated CS-induced mechanical hyperalgesia and allodynia. These results suggest that the pain observed in patients with CFS and FMS involves microglial activation [6], although it remains unclear why microglial accumulation occurs within a restricted area. In the present study, we investigated neuronal activation in specific areas of the spinal cord and dorsal root ganglia (DRG) in rats exposed to CS. Our results suggested that continuous and specific hyperactivation of proprioceptors triggers microglial activation, thereby inducing prolonged abnormal levels of pain.

## Methods

### Experimental animals

A total of 70 male Sprague-Dawley (SD) rats (SLC, Hamamatsu, Japan) were used in this study. All rats were housed in individual cages under a 12-h light-dark cycle at 23 ± 1 °C and 50% relative humidity, with food and water available ad libitum. All rats were acclimatized for at least 1 week prior to the experiment and were maintained in accordance with the *Guide for the Care and Use of Laboratory Animals* [15]. The study was approved by the local animal ethics committee in accordance with the regulations for animal experiments at Nagoya University and Aichi Medical University, the Animal Protection and Management Law of Japan (No. 105), and the Ethical Issues of the International Association for the Study of Pain [16].

### CS animal model

Rats were randomly assigned to CS or no-CS (NCS) control groups. For the CS model, 8-week-old rats were transferred to cages filled with water (23 ± 1 °C) to a height of 1.5 cm for 1–6 days (although rats in our previous study were subjected to 5 days of stress loading, we extended stress loading by 1 day) [6].

CS rats were transferred to cages with water, and the water was exchanged everyday [11]. NCS rats were transferred to ordinary breeding cages. Both CS and NCS rats were maintained in the cages for 1–6 days. Body weight increased each day in the NCS rats only. Previous studies have revealed no significant differences in blood glucose concentration or rectal temperature between the two groups [9]. Behavior and sleep-wake states have also been previously described [9].

### Behavioral pain test

Behavioral testing was performed as previously described [6]. All behavioral tests were performed by blinded investigators. The mechanical paw withdrawal threshold (PWT) of the skin over the hindpaw plantar surface was measured using von Frey filaments (North Coast Medical, San Jose, CA, USA) (i.e., von Frey test (VFT)). The rats were restrained with a jacket around the trunk, although their legs remained freely moveable, and they were treated gently during the experiments. The filament was applied 10 times in ascending order of force (0.16, 0.4, 0.6, 1, 1.4, 2, 4, 6, 8, and 10 g) at intervals of 5 s. The threshold was determined via the method of limits (i.e., increasing and decreasing the force of stimulation). When an animal exhibited at least one withdrawal response among trials but did not exhibit withdrawal at the next lowest filament grade, the former (higher) filament force was regarded as a positive response [6, 17].

To investigate the magnitude and time course of muscular mechanical hyperalgesia, we measured the withdrawal threshold to pressure stimuli (i.e., pressure pain test (PPT)) using an electronic von Frey anesthesiometer (No. 2391; IITC Inc., Los Angeles, CA, USA) [18]. A cone-shaped pusher with a rounded tip (diameter 6 mm; made in our laboratory) was applied to the belly of the lower hind leg extensors, including the tibialis anterior (TA), through shaved skin. The intensity of pressure at which an escape reaction was observed was defined as the withdrawal threshold. Training sessions were carried out for at least four consecutive days. Measurements were performed five times at 30 s intervals, and the mean value (excluding minimum and maximum values) was regarded as the nociceptive threshold.

### Reverse transcription polymerase chain reaction

The plantar skin and TA muscles were removed from CS and NCS rats and quickly frozen in liquid nitrogen. At least five rats were used in each group. Total RNA was purified using the acid guanidine isothiocyanate/phenol/chloroform method and converted to cDNA using SuperScript III (Invitrogen, Carlsbad, CA, USA). Amplification was performed as follows: glyceraldehyde-3-phosphate dehydrogenase (GAPDH) (sense: 5′-CTACATGGTCTACATGTTCCAGTATG-3′, antisense: 5′-AGTTGTCATGGATGACCTTGG-3′; 26 cycles), interleukin-6 (IL-6) (sense: 5′-GGATACCACCCACAACAGAC-3′, antisense: 5′-CATTGGAAGTTGGGGTAGGA-3′; 34 cycles), interleukin-1β (IL-1β) (sense: 5′-GTGTCTGAAGCAGCTATGGC-3′, antisense: 5′-TCCTTGTACAAAGCTCATGG-3′; 32 cycles for TA and 34 cycles for plantar skin), and tumor necrosis factor-α (TNF-α) (sense: 5′-CCACGCTCTTCTGTCTACTG-3′, antisense: 5′-CCTCGTCCCTTGAAGAGAAC-3′; 32 cycles for TA and 34 cycles for plantar skin), at 94 °C for 30 s, 60 °C for 30 s, and 72 °C for 30 s. The amplified products were electrophoresed on 1.0% agarose gels and stained with ethidium bromide. For the positive control of inflammation, complete Freund’s adjuvant (CFA) (100 μl) was injected into the crural muscles and plantar skin.

### Immunohistochemistry

Following behavioral testing, animals were anesthetized via an intraperitoneal injection of sodium pentobarbital (45 mg/kg) and transcardially perfused with saline followed by Zamboni’s fixative (2% paraformaldehyde with 0.2% picric acid in 0.1 M phosphate buffer). The L1–6 spinal cord segment (SC), bilateral L1–6 DRG, plantar skin, and soleus muscle were immediately dissected and post-fixed in the same fixative overnight at 4 °C, followed by immersion in 30% sucrose in 0.1 M PBS at 4 °C for 2 days. The tissues were then frozen in liquid nitrogen and cooled in isopentane, following which they were processed for sectioning using a cryostat. The tissues were cut into serial sections using a cryostat (SC 20-μm thickness; DRG, skin, and muscle 10-μm thickness), and the sections were thaw-mounted on Superfrost Plus microscope slides (Matsunami, Tokyo, Japan). Sections were blocked with 1% BSA and 0.3% Triton-X100 (Sigma-Aldrich, St. Louis, MO, USA), following which they were incubated with the appropriate primary antibody overnight at 4 °C. Primary antibodies used for immunohistochemistry are listed in the supporting information (Additional file 1: Table S1). Sections were then incubated with the appropriate secondary antibody for 90 min at room temperature: Alexa 488 donkey anti-rabbit immunoglobulin G (IgG) (1:1000) or Alexa 594 donkey anti-rat IgG (1:1000) (Molecular Probes, Invitrogen). Sections were coverslipped using a standard mounting medium and visualized via fluorescence microscopy using a × 10 objective (BZ9000, Keyence, Osaka, Japan).

### Quantification of ATF3-positive DRG neurons

To investigate changes in the expression of ATF3 (a marker for damaged/hyperactivated neurons) during stress loading, we calculated the ratio of ATF3-positive cells to the total number of neurons per section, which was determined using DAPI (nuclear staining). We analyzed an average of three to four sections (L2–6 DRG) per animal. On microscopic images, dots were entered into positive cells using Photoshop CC (Adobe, San Jose, CA, USA), and the number of cells was counted using ImageJ 1.50i (NIH, Bethesda, MD, USA). Using the same methods, we further investigated subtypes of neurons expressing ATF3 by calculating co-expression of TrkA, IB4, and CGRP (markers for nociceptive neurons); TrkB (a marker for mechanoreceptive neurons); and TrkC and VGluT1 (markers for proprioceptive neurons).

### Retrograde neuron labeling

DRG neurons and spinal motor neurons innervating the relevant muscles were identified using retrograde fluorogold (FG) labeling. Under inhalation anesthesia with 2% isoflurane, the skin of the hindlimb was incised to expose the muscle. FG (5 μl at 4%; Fluorochrome, LLC, Denver, CO, USA) in saline was injected into each muscle of the hindlimb (soleus, gastrocnemius, TA, flexor digitorum superficialis, flexor digitorum brevis, peroneus longus, extensor digitorum longus, lumbricalis). To prevent FG from penetrating other muscles, the muscle surface was thoroughly washed with saline after injection. The skin was then sutured, and the CS model was prepared 7 days after FG injection.

### Electromyography recording

For electromyography (EMG) recording using the telemetry system, surgery was performed on rats under anesthesia induced with 2% isoflurane. The muscle was exposed via a small skin incision on the left lower leg, and two insulated stainless steel wire EMG electrodes (AS631; Cooner Wire, Chatsworth, CA) were implanted into the left soleus via a small needle hole. The electrode wire was tunneled subcutaneously through an incision along the anterior surface of the thigh. The telemeter transmitter (IMG-20EMr, Star Medical, Tokyo Japan) was placed in the peritoneal cavity. The rats recovered for at least 7 days prior to stress loading. Baseline EMG activity of the soleus muscle was recorded 1 day prior to stress loading, and further recordings were performed on day 6 of stress loading. Data were stored on a PC (Windows 7 Professional). EMG signals were rectified using LabChart software (v8.1.5 for Windows, ADInstruments, Sydney, Australia), following which data were normalized by calculating the integrated value for 6 h for each day (12:00–18:00) and night (0:00–6:00).

### Ankle arthrodesis

Under inhalation anesthesia with 2% isoflurane, an incision was made in the plantar skin of the right hindpaw, and a hole was drilled through the calcaneus and talus to the medullary cavity of the tibia using a hand drill (drill bit diameter 1 mm; Tamiya, Inc., Shizuoka, Japan). A 21-G injection needle was then inserted from the sole of the foot to the medullary cavity of the tibia. The incised skin was sutured at the conclusion of surgery. In order to exclude the influence of ankle arthrodesis, a CS model was developed 6 weeks after surgery.

### Quantification of activated microglia

Three tissue sections from each L5 spinal cord segment of the ankle arthrodesis CS model were randomly selected, and the number and area of Iba1-positive cells were measured by fluorescence microscopy. Two observation fields (the medial region of the dorsal horn field and the dorsal area of the ventral horn field) of each 10,000 μm^2^ were selected and captured using a digital camera attached to the fluorescence microscope (BZ9000, Keyence). The total area occupied by the Iba1-positive microglia and number of Iba1-positive microglial cells within the two fields were quantified using the image processing and analyzing software, Image J (NIH). The mean values of the number and area of Iba1-positive cells in both sides were obtained using three SC sections from each of four animals.

### Statistical analyses

All data are expressed as the mean ± standard error of the mean (SEM). PWTs for muscle pain were analyzed via a two-way, repeated-measures analysis of variance (ANOVA), followed by the Holm-Sidak multiple comparisons test. Data from the VFT were analyzed using a non-parametric two-way, repeated-measures rank ANOVA, followed by the Holm-Sidak multiple comparisons test. Data for ATF3-positive DRG neurons were analyzed via a one-way ANOVA with Tukey’s test. EMG data were analyzed via a one-way ANOVA. The level of statistical significance was set at *p* < 0.05. See figure legends for further details. Data for Iba1-positive microglial cells were analyzed via a parametric paired *t* test.

## Results

### Six days of stress loading substantially prolongs pain behaviors

**Fig. 1:**
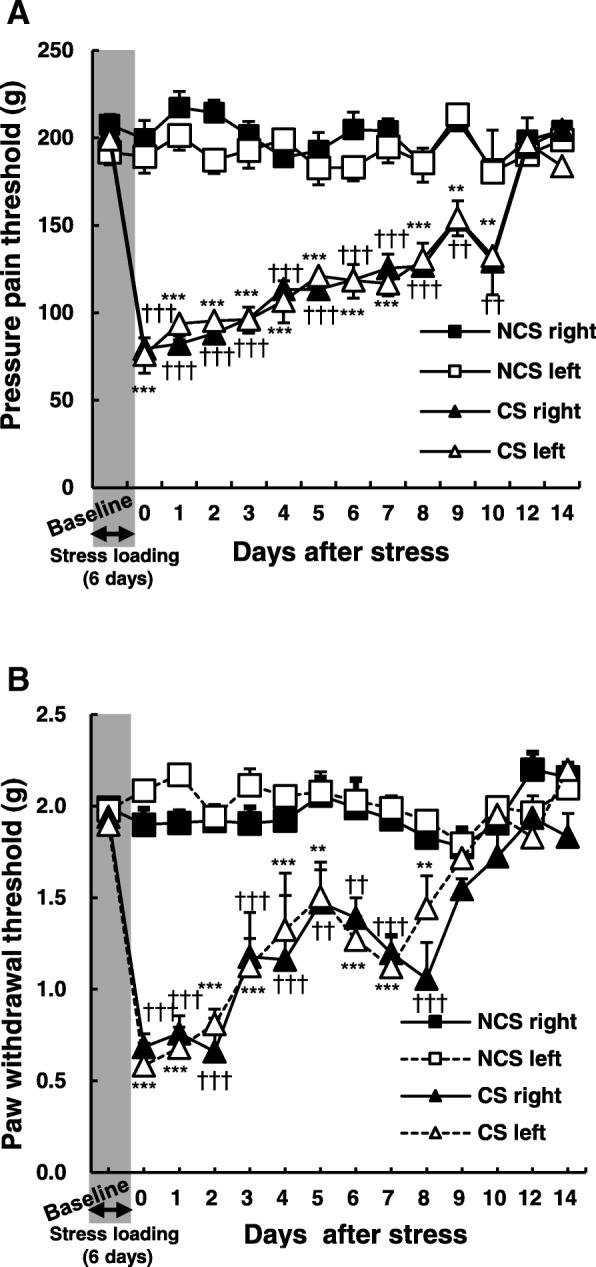
Six days of stress loading significantly prolonged pain behaviors. **a** Pressure pain threshold of the muscle. **b** Mechanical withdrawal threshold of the skin (von Frey test (VFT)). Note the significant decreases in pain threshold for 10 days (pressure pain threshold test) and skin pain for 8 days (VFT). NCS, no continuous stress; right, right leg or paw; left, left leg or paw. *n* = 5 (each group); **p < 0.01, ***p < 0.001 vs. NCS left; ††*p* < 0.01, †††*p* < 0.001 vs. NCS right. Two-way repeated measures ANOVA followed by the Holm-Sidak multiple comparisons test

Previously, we developed our CS model using 5 days of stress loading, following which the CS rats exhibited tactile allodynia and mechanical hyperalgesia in the bilateral hindlimbs. In the model, significant decreases in PWT were observed for 2 days after CS, and this response was restored to the control level on the third day after CS. In the PPT, the muscle pain pressure threshold was significantly decreased immediately after CS and maintained for the following 4 days [6]. When we extended stress loading by 1 day (i.e., 6 days of stress loading), the durations of allodynia and mechanical hyperalgesia were dramatically extended to 10 days and 8 days, respectively (Fig. 1a, b).

### Continuous stress activates proprioceptors in the DRG

**Fig. 2:**
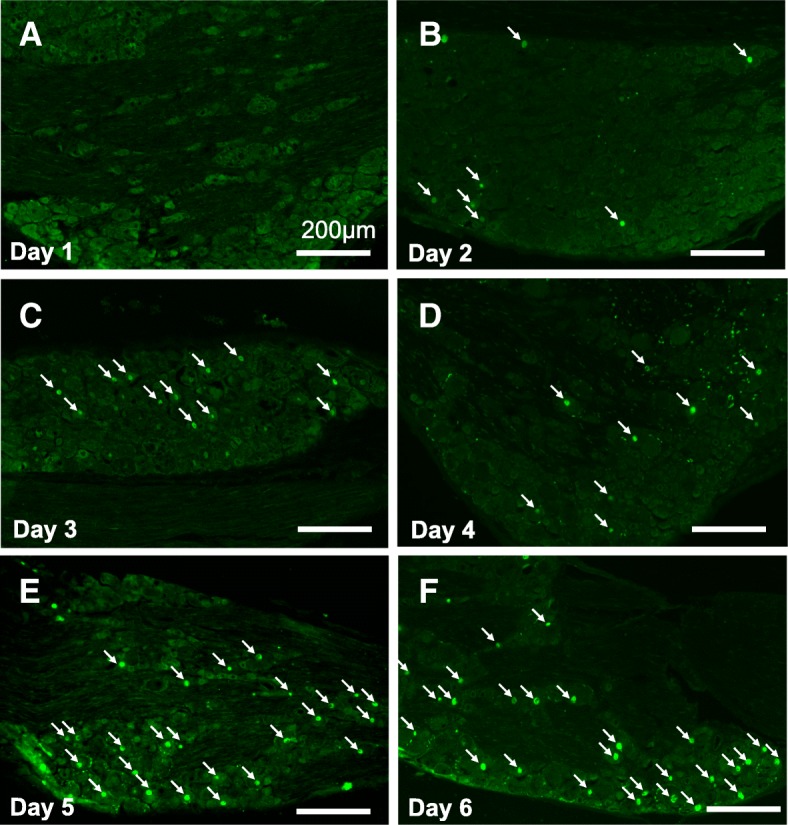
Appearance of ATF3-positive signal in L5 dorsal root ganglion (DRG) neurons during stress loading. **a–f** ATF3 immunoreactivity was first observed in the L5 DRG at day 2 (**b**). The number of the ATF3-positive cells increased as CS progressed (**c–f**). Arrows indicate ATF3-positive nuclei. Scale bar 200 μm

ATF3 expression, a marker of neuronal hyperactivation and injury [19, 20], was examined in the DRG during CS. ATF3-positive neurons were first observed on the second day of CS, and the number of positive cells gradually increased until the termination of stress loading after day 6 (Fig. 2). ATF3 expression was mainly observed at the L5 level of the DRG (Additional file 2: Figure S1).

**Fig. 3:**
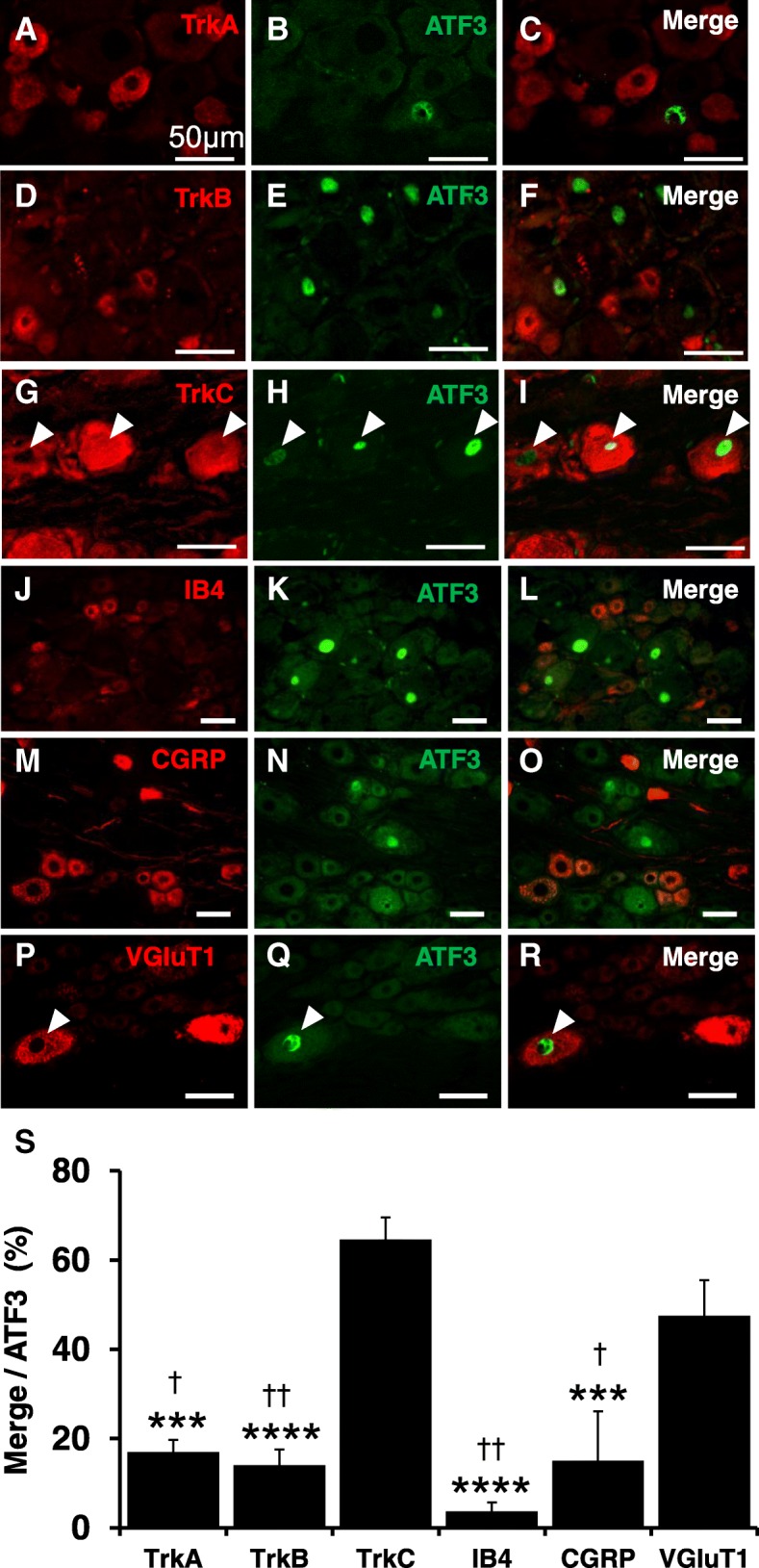
Identification of cell types among ATF3-positive neurons. Double immunofluorescence staining was performed using L5 dorsal root ganglion (DRG) tissues from rats subjected to continuous stress-loading (CS). **a–r** Representative double immunofluorescence labeling for ATF3 (**b**, **e**, **h**, **k**, **n**, **q**) with the markers TrkA (**a**), TrkB (**d**), TrkC (**g**), IB4 (**j**), CGRP (**m**), and VGluT1 (**p**). **c**, **f**, **i**, **l**, **o**, **r** Merged images of the respective two photos on the left. Arrowheads indicate co-expressing cells. Scale bar 50 μm. **s** The co-expression rate of each marker in ATF3-positive cells. *n* = 4. ***p < 0.001 vs. TrkC. ****p < 0.0001 vs. TrkC. †*p* < 0.05 vs. VGluT1. ††p < 0.01 vs. VGluT1. One-way ANOVA with Tukey’s test

To identify the types of sensory neurons expressing ATF3 in the DRG, we examined co-expression of ATF3 with TrkA, TrkB, TrkC, IB4, CGRP, and VGluT1. Among these, more than 60% of ATF3-positive cells co-expressed TrkC, a marker of proprioceptive neurons, while approximately 50% co-expressed VGluT1. Rates of co-expression with TrkA, TrkB, IB4, and CGRP were less than 20%, suggesting that most ATF3-expressing cells in the DRG were proprioceptive neurons (Fig. 3).

### ATF3-positive DRG neurons project to the soleus

**Fig. 4:**
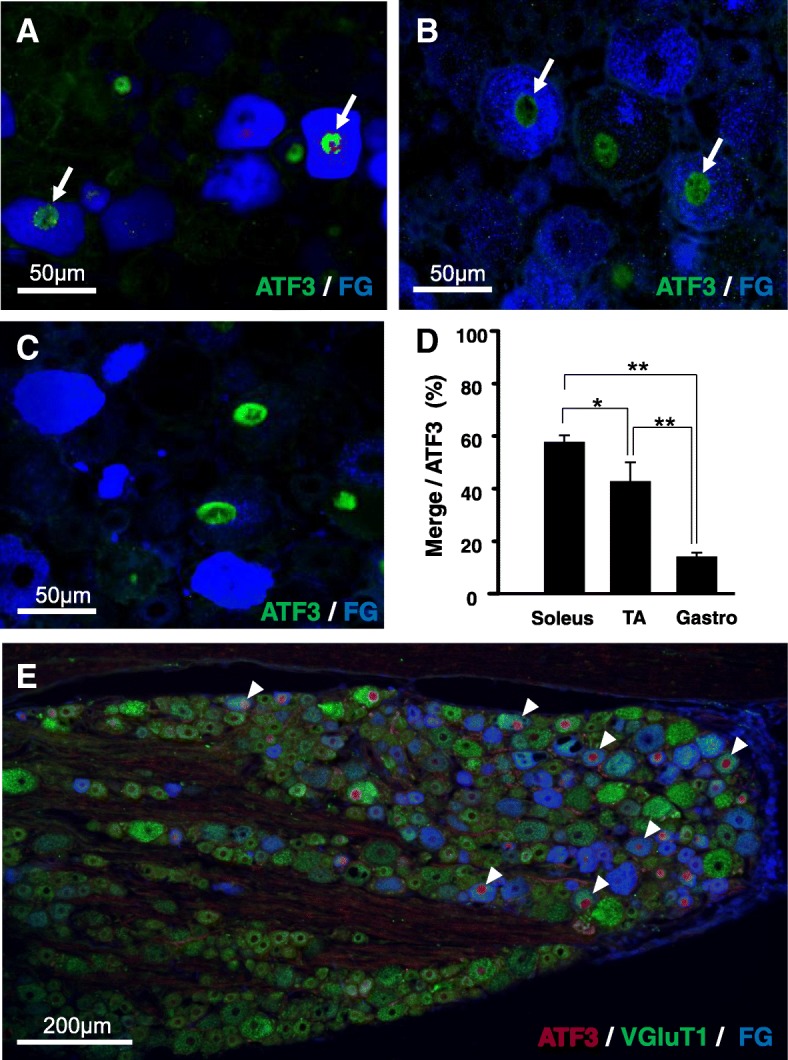
A significant number of ATF3-positive dorsal root ganglion (DRG) neurons projected to the soleus. **a–c** Double staining for ATF3 (green) and fluorogold (FG)-labeled (blue) neurons in the L5 DRG of rats subjected to continuous stress-loading (CS). FG was injected into the soleus (**a**), tibialis anterior (**b**), or gastrocnemius (**c**). **d** The proportion of FG-labeled neurons among ATF3-positive cells after FG injection: Soleus, 57.7 ± 7.3%; tibialis anterior, 42.7 ± 14.7%; and gastrocnemius, 14.0 ± 4.1%. Soleus (*n* = 8), tibialis anterior (*n* = 4), and gastrocnemius (*n* = 6). *p < 0.05, **p < 0.01. One-way ANOVA with Tukey’s test. **e** A representative photo of the triple labeling for VGluT1 (green), ATF3 (red), and FG (blue) in the L5 DRG of CS rats. Arrowheads indicate co-expression

We then examined the innervation of the ATF3-positive L5 DRG neurons via retrograde labeling (Fig. 4). We injected FG into regions of possible innervation such as the soleus, gastrocnemius, TA, flexor digitorum superficialis, flexor digitorum brevis, peroneus longus, extensor digitorum longus, and lumbricalis. A total of 57.7 ± 7.3% of the ATF3-positive neurons were simultaneously labeled by FG when the tracer was injected into the soleus; however, such high rates of FG labeling were not observed in other regions (Fig. 4d). Triple labeling with anti-VGluT1 (a proprioceptive marker), anti-ATF3 (a marker of neuronal hyperactivation), and FG (a retrograde tracer) was observed in L5 DRG neurons (Fig. 4e, arrowheads), suggesting that many ATF3-positive DRG neurons were associated with proprioception in the soleus.

### Appearance of microglial accumulation and activation in a restricted dorsal region of the ventral horn

**Fig. 5:**
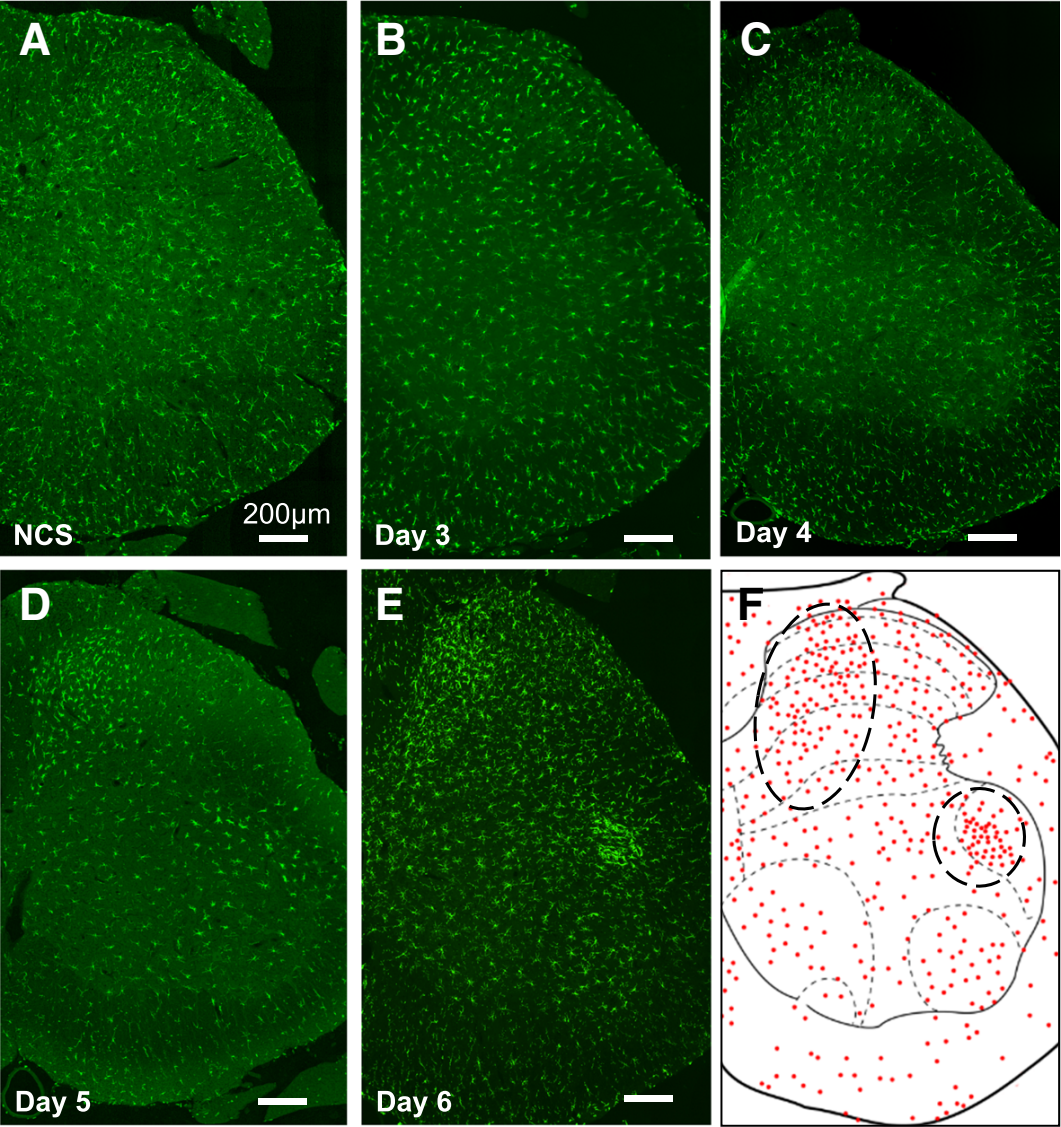
Profile of microglial activation and accumulation during continuous stress-loading (CS) in the L5 spinal cord. **a–e** Iba-1 positive cells (microglia) in the L5 spinal cord in the no-CS (NCS) (**a**) group and at 3 days (**b**), 4 days (**c**), 5 days (**d**), and 6 days (**e**) after the initiation of CS. **f** A representative dot plot indicating microglial localization in the L5 spinal cord during 6 days of CS. Areas in which microglial activation and accumulation were observed are surrounded by black dotted lines. Scale bar 200 μm

**Fig. 6:**
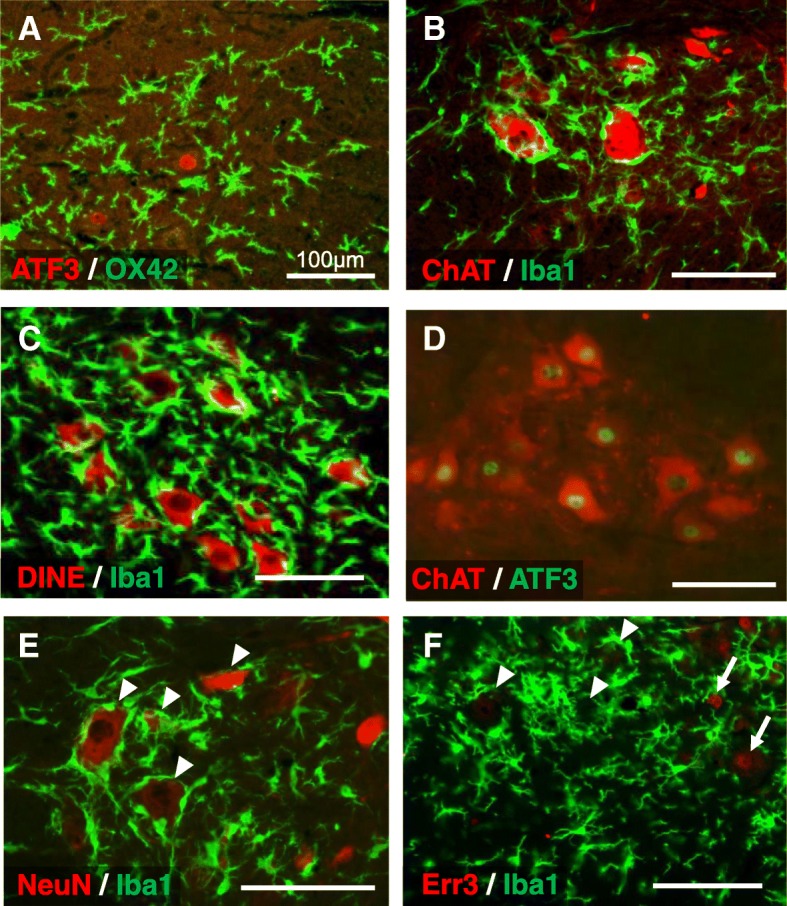
Neurons surrounded by activated microglia after 6 days of CS were α-motor neurons. **a–f** Double labeling images of the dorsal region of the ventral horn of the L5 spinal cord on day 6 of CS. **a** OX42 (activated microglia, green)/ATF3 (red). **b** Iba1 (microglia, green)/ChAT (motor neurons, red). **c** Iba1 (microglia, green)/DINE (motor neurons, red). **d** ATF3 (green)/ChAT (motor neurons, red). **e** Iba1 (microglia, green)/NeuN (α-motor neurons, red). **f** Iba1 (microglia, green)/Err3 (nuclear staining of γ-motor neurons, red). Arrowheads indicate α-motor neurons, while arrows indicate γ-motor neurons. All scale bars 100 μm

As described in our previous study [6], microglial activation and accumulation were observed in the medial region of the dorsal horn after 5 days of CS (Fig. 5a–d). Intriguingly, microglial activation and accumulation were also observed in the dorsal area of the ventral horn when the duration of CS was extended to 6 days (Fig. 5e, f). The accumulated microglia in the dorsal part of the ventral horn exhibited relatively flat cell bodies with short and thick processes, indicative of an activated state (Fig. 6a), and appeared surrounding relatively large neurons (Fig. 6b). Importantly, the activation patterns observed in the dorsal horn and the ventral horn were distinct; microglial adhesion to the neuronal soma was specifically observed in the ventral horn. In addition, large ChAT-positive neurons, which were assumed to be motor neurons, were surrounded by Iba1-positive microglia (Fig. 6b). This result was also confirmed using another motor neuron marker, DINE (Fig. 6c) [21–23]. Some ChAT-positive neurons located in this region also expressed ATF3 (Fig. 6d), suggesting that a subset of ATF-3 positive motor neurons located in a restricted dorsal region of the ventral horn is surrounded by microglia following the induction of CS. Next, we examined whether these motor neurons were α- or γ-type motor neurons using antibodies against NeuN and Err3, respectively. As seen in Fig. 6e and f, cells surrounded by microglia were NeuN-positive (arrowheads in Fig. 6e) but not Err3-positive (arrows in Fig. 6f), suggesting that microglia surround α-motor neurons within a restricted dorsal region of the ventral horn.

### Motor neurons surrounded by microglia project to the soleus

**Fig. 7:**
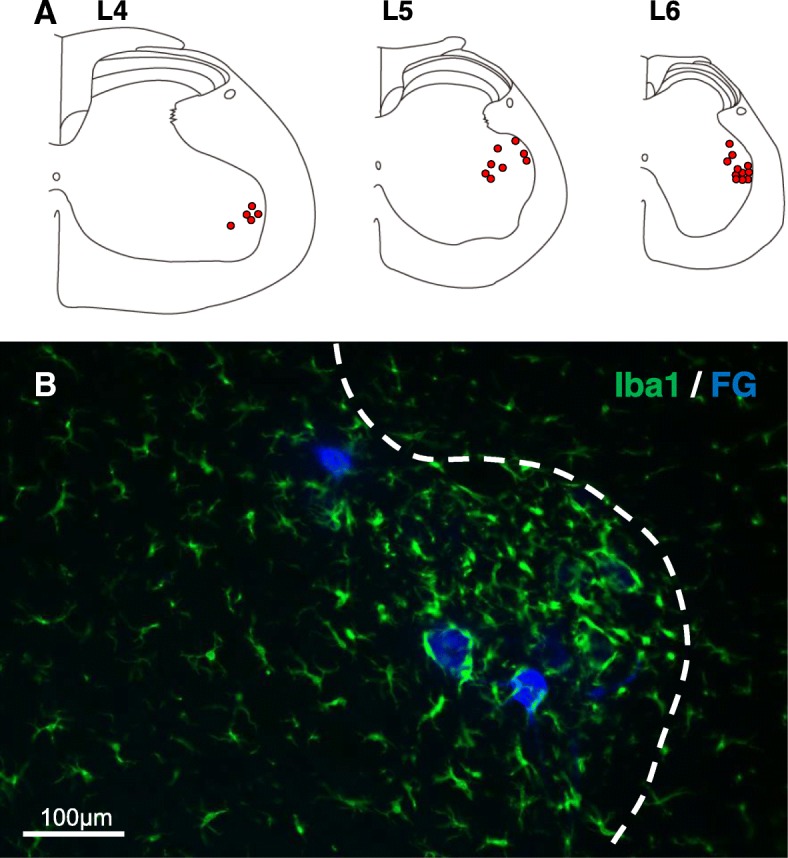
Motor neurons surrounded by microglia projected to the soleus. **a** A map indicating the localization of retrogradely labeled FG-positive cells after FG injection into the soleus. The FG-positive motor neurons are plotted on a standardized chart. **b** FG (blue)-positive motor neurons were attracted to Iba1-positive microglia (green) at L5. The white dotted line in **b** indicates the border between white matter and gray matter

**Fig. 8:**
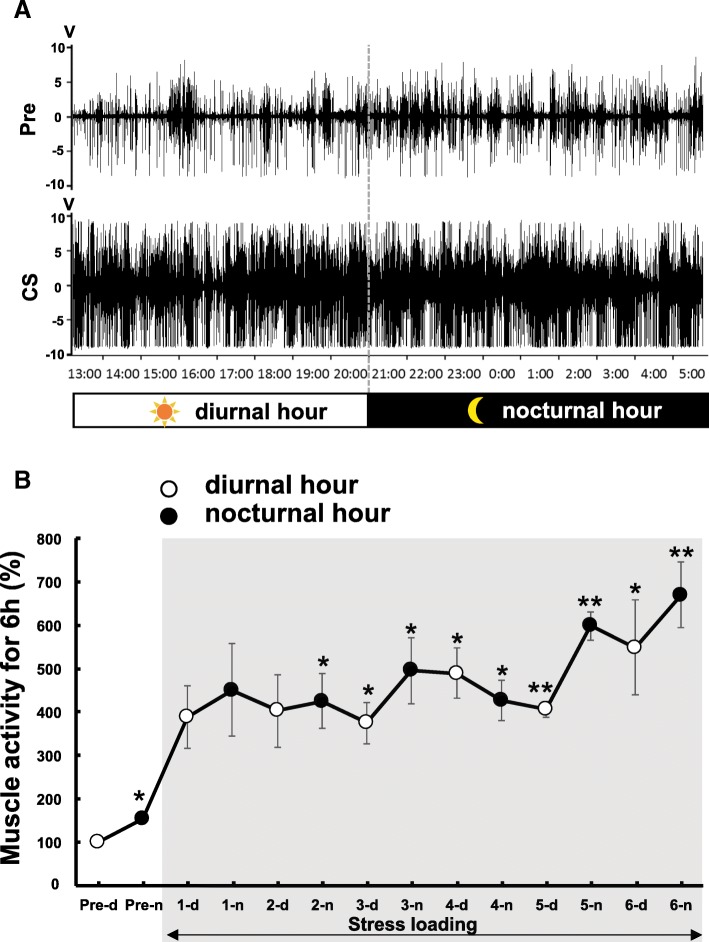
Electromyogram of the soleus muscle during stress loading. **a** A representative chart of electromyographic activity for the no-CS (NCS/Pre) and CS groups on day 6 of CS. **b** Profile of muscle activity prior to and during stress loading. Muscle activity was obtained via the integration of spike power for 6 diurnal hours (12:00–18:00) and 6 nocturnal hours (0:00–6:00) for each CS day. The activity observed during the 6 diurnal hours (12:00–18:00) of the pre-stress-loading period (Pre-d) was defined as 100%. Pre-n, the 6 nocturnal hours (0:00–6:00) of activity prior to stress loading. All points represent the average +/− standard error of the mean (SEM) from four animals. White circle, % index of diurnal muscle activity; black circle, % index for nocturnal muscle activity. *n* = 4. *p < 0.05, **p < 0.01 vs. Pre-d. One-way ANOVA

We then examined the target muscle of the α-motor neurons that were surrounded by microglia at 6 days after CS. We first identified FG-positive neurons via retrograde labeling. FG-labeled motor neurons were dominantly localized in the dorsal region of the ventral horn at L4–6 (Fig. 7a), where microglial accumulation and ATF3-positive neurons were observed, when FG was injected into the soleus. Double labeling with FG and Iba1 was also observed when FG was injected into the soleus, indicating that FG-positive motor neurons were surrounded by activated microglia (Fig. 7b). These results indicated that a subset of α-motor neurons that project to the soleus is highly activated by CS and that chronic activation of motor neurons may attract microglia. To investigate this possibility, we then collected EMG data for the soleus using a telemetry system (Fig. 8). EMG activity was two- to threefold higher in the CS group than in the control group, and this increase in EMG activity was observed both nocturnally and diurnally, gradually increasing as CS progressed (Fig. 8). Despite such changes, no soleus damage or additional macrophage infiltration into the muscle was observed (Additional file 3: Figure S2A–D). No concomitant increases in inflammatory cytokine expression were observed in the soleus tissue of CS rats (Additional file 3: Figure S2E). These results indicate that CS elicits hyperactivation of the soleus during stress loading without apparent damage to the muscle tissue.

### Immobilization of the soleus attenuates ATF3 expression and microglial accumulation after CS

**Fig. 9:**
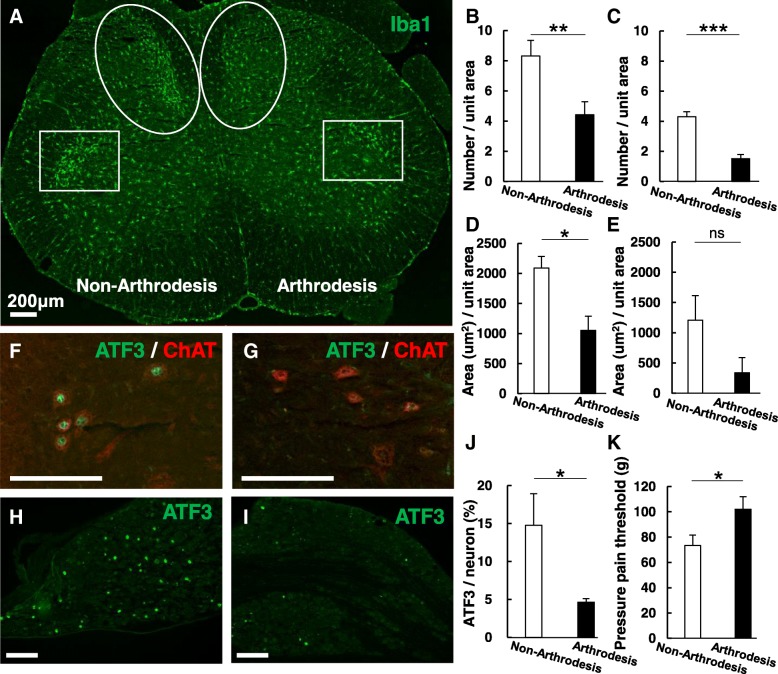
Ankle joint immobilization suppressed microglial activation. **a** Unilateral arthrodesis of the right side was performed to immobilize the ankle joint (i.e., left = non-arthrodesis side). Clear microglial accumulation was observed in the L5 spinal cord in the medial region of the dorsal horn and the dorsal region of the ventral horn (non-arthrodesis side), whereas no such accumulation was observed on the arthrodesis side (arthrodesis side). **b** Quantification of microglial cell number/unit area (10,000 μm^2^) in the medial region of the L5 dorsal horn. **c** Quantification of microglial cell number/unit area (10,000 μm^2^) in the dorsal region of the L5 ventral horn. **d** Quantification of microglial occupied area/unit area in the medial region of the L5 dorsal horn. **e** Quantification of microglial occupied area/unit area in the dorsal region of the L5 ventral horn. **f** ATF3 immunoreactivity (green) was observed in ChAT-positive cells (motor neurons, red) in the rectangular inset region of the non-arthrodesis side in **a**. **g** ATF3 immunoreactivity was not observed in ChAT-positive cells (motor neurons, red) in the inset region of the arthrodesis side in **a**. **h** Expression of ATF3 (green) was observed in the L5 dorsal root ganglion (DRG) of the non-arthrodesis side. **i** Expression of ATF3 was suppressed in the L5 DRG of the arthrodesis side. Scale bar 200 μm. **j** ATF3 expression was significantly suppressed in the L5 DRG of the arthrodesis side. **k** Changes in the pressure pain threshold of the muscle after CS on the non-arthrodesis and arthrodesis sides. The pain threshold increased on the arthrodesis side. *n* = 4. *p < 0.05. **p < 0.01. ***p < 0.001. Paired *t* test

The soleus is an antigravity muscle that becomes overactivated during CS, thereby inducing hyperactivation of the proprioceptive neurons in the DRG that innervate muscle spindles in the soleus. Thus, microglial activation along the central branches of proprioceptive DRG neurons in the dorsal horn is likely to elicit chronic pain. Attenuating the hypertonic activity of the soleus may therefore attenuate CS-induced pain and microglial activation. To investigate this possibility, we attempted to suppress the ankle joint movement via arthrodesis. Following 6 weeks of recovery after the ankle fixation operation, the rats were subjected to 6 days of CS. Ankle joint immobilization significantly suppressed the accumulation of microglia in the ipsilateral dorsal and ventral horns, whereas significant accumulation of microglia was observed in both the dorsal and ventral horns on the contralateral side (Fig. 9a–e). Ankle immobilization concomitantly decreased the expression of ATF3 in ipsilateral motor and DRG neurons (Fig. 9f–j). Significant increases in PPT during CS were also observed on the side of the arthrodesis after fixation (Fig. 9k).

## Discussion

The results of the present study demonstrate that the rat models of CFS experience long-lasting allodynia and muscle pain even in the absence of peripheral inflammation and nerve injury and that this pain is likely to be initiated by continuous hyperactivation of proprioceptors in the DRG. Such long-lasting proprioceptor activation would continuously stimulate the reflex arc in the spinal cord, further inducing microglial activation along the arc. Microglial activation would in turn exacerbate and prolong the abnormal pain [24–29], suggesting that proprioceptor-induced microglial activation may underlie pain generation in CFS and FMS.

In our previous study, in which CS was conducted for 5 days, significant decreases in PWT were observed for 2 days following stress loading, along with increases in muscular pain/hyperalgesia for 4 days [6]. However, extending stress loading by an additional day in the present study dramatically prolonged the duration of pain: 10 days in the VFT and 8 days in the PPT. Although the reason for such increases in pain duration remains unknown, the extent of microglial accumulation and activation after 6 days of CS appears much higher in both the dorsal and ventral horns than that after 5 days (Fig. 5d, e). These results suggest that the duration of pain is associated with the extent of microglial activation.

In the present chronic fatigue model, three notable alterations were observed in different regions: ATF3 expression in DRG neurons, microglial accumulation in the dorsal horn, and microglial adhesion to motor neurons in the ventral horn. Interestingly, these events occurred in a sequential manner: ATF3 expression in DRG neurons was observed after 2 days of CS, activation and accumulation of microglia in the dorsal horn were observed after 4 days of CS, and microglial accumulation and adhesion to motor neurons were observed after 6 days of CS. Our results further indicated that ATF3-positive neurons in the DRG were predominantly proprioceptors, more than half of which innervated the soleus, an antigravity muscle. The dorsomedial area of the dorsal horn in which microglial accumulation was observed represents the area through which proprioceptive primary afferent fibers pass and the area containing the ATF3-positive motor neurons that were surrounded by microglia, whose axons projected to the soleus. These results indicate that sequential activation occurs along the reflex arc of the spinal cord and that chronic activation of this circuit may activate microglia along the circuit, in turn leading to and/or prolonging chronic pain [6, 30, 31]. In terms of sleep situation, the rats may sleep by leaning against the cage wall [9]; however, this posture may chronically induce lower limb tone, particularly that of the soleus, which is a slow-twitch muscle. This may cause chronic proprioceptive hyperactivation in the soleus, which has a significant number of muscle spindles. Our animal model results, thus, may indicate that patients with CFS and FMS may initially experience unconscious and prolonged activation of muscle tonus that causes hyperactivation of proprioceptors in the DRG, and that this prolonged activation leads to the stimulation of microglia along the proprioceptor-mediated reflex arc in the spinal cord. The activated microglia may then become so-called memory cells of pain, and patients may feel pain as long as the activated microglia exist in the spinal cord. Acute increases in muscle activity can trigger pain in patients with FMS, which may be explained by proprioceptor activation leading to hyperactivation of the reflex arc. As minocycline suppresses microglial activation and chronic pain in this model [6], such efforts may be crucial for suppressing the microglial activity in CFS and FMS. In addition, reducing muscle tonus by manual therapy may be effective in patients with CFS and FMS.

In rats, the lower limbs are primarily important for changing and maintaining posture, and the duration of the standing posture is markedly prolonged in the water cage [9, 10]. EMG recordings from the present study revealed continuously high soleus activity during stress loading (Fig. 8), suggestive of proprioceptor activation, which may explain why many proprioceptive neurons in the L5 region expressed ATF3. Notably, no signs of tissue damage such as inflammation or nerve injury were observed in the leg muscles or plantar skin of CS rats. Among the leg muscles, the soleus is known to have a higher density of muscle spindles. Previous rat studies have reported that the density of muscle spindles is six to eight times higher in the soleus than in the gastrocnemius or flexor hallucis longus [32]. The human soleus contains the most sensitive muscle spindles, which may explain why the anatomical density of spindle afferents is over twice as high in the soleus than in the gastrocnemius [32, 33]. Because the soleus muscle is monoarticular and its passive fascicle length changes depending only on the angle of the ankle joint, the soleus may be one of the most important muscles for maintaining posture [33]. These findings may explain why the proprioceptors innervating the soleus are so sensitive to CS and dominantly expressed ATF3 under such conditions.

## Conclusions

In conclusion, the present study revealed that CS activates the neuronal pathway along the proprioceptor-mediated spinal reflex arc and that overactivation of this arc activates microglia along the arc. Such proprioceptor-induced microglial activation may play a key role in the initiation and maintenance of abnormal pain in patients with CFS and FMS, although significant studies are necessary before extrapolating those findings to patients with CFS and FMS.

## Additional files

Additional file 1: **Table S1.** Characterization of the primary antibodies used for immunohistochemistry (IHC) (PDF 90 kb)

Additional file 2: **Figure S1.** Expression profile of ATF3-positive neurons in the dorsal root ganglion (DRG). A: ATF3 was hardly observed in the L5 DRG of control rats (NCS). B–F: ATF3-positive neurons in each DRG (L2–6) were observed after 6 days of continuous stress-loading (CS). Scale bar: 200 μm. G: Rates of ATF3-positive cells per section were measured from L5 sections in the NCS group and L2-L5 sections in the CS group. Note that the rate of ATF3 expression peaked in the L5 DRG and was significantly higher than that in the L5 DRG of NCS rats. *n* = 5. ***p < 0.001. Mann-Whitney *U* test (PDF 519 kb)

Additional file 3: **Figure S2.** No evidence of inflammation was observed in the soleus or planter skin. A–B: Expression of the macrophage markers OX42 (A and C) and Iba1 (B and D) was examined, although no macrophage accumulation was observed in the soleus of CS rats. Scale bar: 100 μm. E: Polymerase chain reaction (PCR) analysis was used to examine the mRNA expression of representative inflammatory cytokines. No increases in cytokine expression were observed in the CS group relative to the expression in the NCS group, although CFA injection revealed marked increases in mRNA expression in both groups (PDF 474 kb)

## Abbreviations

ANOVA: Analysis of variance
CFA: Complete Freund’s adjuvant
CFS: Chronic fatigue syndrome
CS: Continuous stress-loading
DRG: Dorsal root ganglia
EMG: Electromyography
FG: Fluorogold
FMS: Fibromyalgia syndrome
FSS: Functional somatic syndrome
GAPDH: Glyceraldehyde-3-phosphate dehydrogenase
GHRH: Growth hormone-releasing hormone
IBS: Irritable bowel syndrome
IL-1β: Interleukin-1β
IL-6: Interleukin-6
PPT: Pressure pain test
PWT: Paw withdrawal threshold
RT-PCR: Real-time polymerase chain reaction
SC: Spinal cord segment
SD: Sprague-Dawley
SEM: Standard error of the mean
TA: Tibialis anterior
TNF-α: Tumor necrosis factor-α
VFT: von Frey test
α-MSH: Alpha-melanocyte stimulating hormone
