# Supplementary material for: Hyperactivation of proprioceptors induces microglia-mediated long-lasting pain in a rat model of chronic fatigue syndrome
Source: J Neuroinflammation. 2019 Mar 30;16:67. doi: 10.1186/s12974-019-1456-x (PMC6441145; doi:10.1186/s12974-019-1456-x)
Supplement: Supplementary file 1 — Table S1. Characterization of the primary antibodies used for immunohistochemistry (IHC) (PDF 90 kb) [file 12974_2019_1456_MOESM1_ESM.pdf]

Additional files

Table S1. Characterization of the primary antibodies used for immunohistochemistry (IHC).

Antibody against	Host	Code, source	Dilution
ATF3	rabbit	sc-188, Santa Cruz, USA	1:1000
Iba1	rabbit	019-19741, Wako, Japan	1:1000
SV2A	rabbit	ab32942, Abcam, USA	1:400
CGRP	goat	1720-9007, Bio-Rad, USA	1:5000
ChAT	goat	AB144P, Merck, Germany	1:200
DINE	goat	sc-11338, Merck , Germany	1:1000
TrkA	goat	AF1056, R&D systems, USA	1:500
TrkB	goat	sc-12, Santa Cruz, USA	1:50
TrkC	goat	AF1404, R&D systems, USA	1:100
Err3	mouse	PP-H6812-00, R&D systems, USA	1:100
NeuN	mouse	MAB377, Santa Cruz, USA	1:100
VGluT1	guinea pig	AB5905, Merck, Germany	1:1000
IB4	--	L2895, Sigma, USA	1:1000
