# Supplementary figures and images for: Hyperactivation of proprioceptors induces microglia-mediated long-lasting pain in a rat model of chronic fatigue syndrome
Source: J Neuroinflammation. 2019 Mar 30;16:67. doi: 10.1186/s12974-019-1456-x (PMC6441145; doi:10.1186/s12974-019-1456-x)

## Additional files

Figure S1

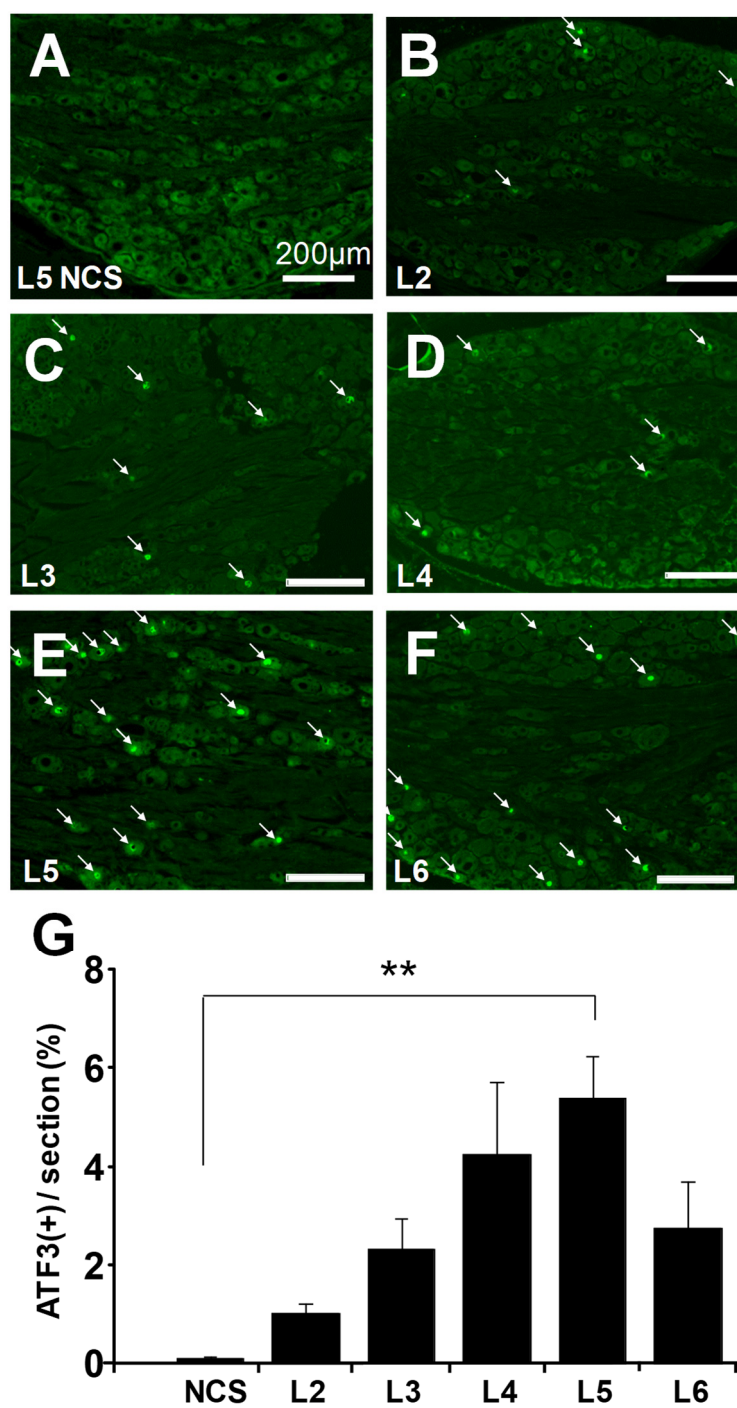

Supplement: Supplementary file 2 — Figure S1. Expression profile of ATF3-positive neurons in the dorsal root ganglion (DRG). A: ATF3 was hardly observed in the L5 DRG of control rats (NCS). B–F: ATF3-positive neurons in each DRG (L2–6) were observed after 6 days of continuous stress-loading (CS). Scale bar: 200 μm. G: Rates of ATF3-positive cells per section were measured from L5 sections in the NCS group and L2-L5 sections in the CS group. Note that the rate of ATF3 expression peaked in the L5 DRG and was significantly higher than that in the L5 DRG of NCS rats. n = 5. ***p < 0.001. Mann-Whitney U test (PDF 519 kb) [file 12974_2019_1456_MOESM2_ESM.pdf]

Additional files

Figure S2.

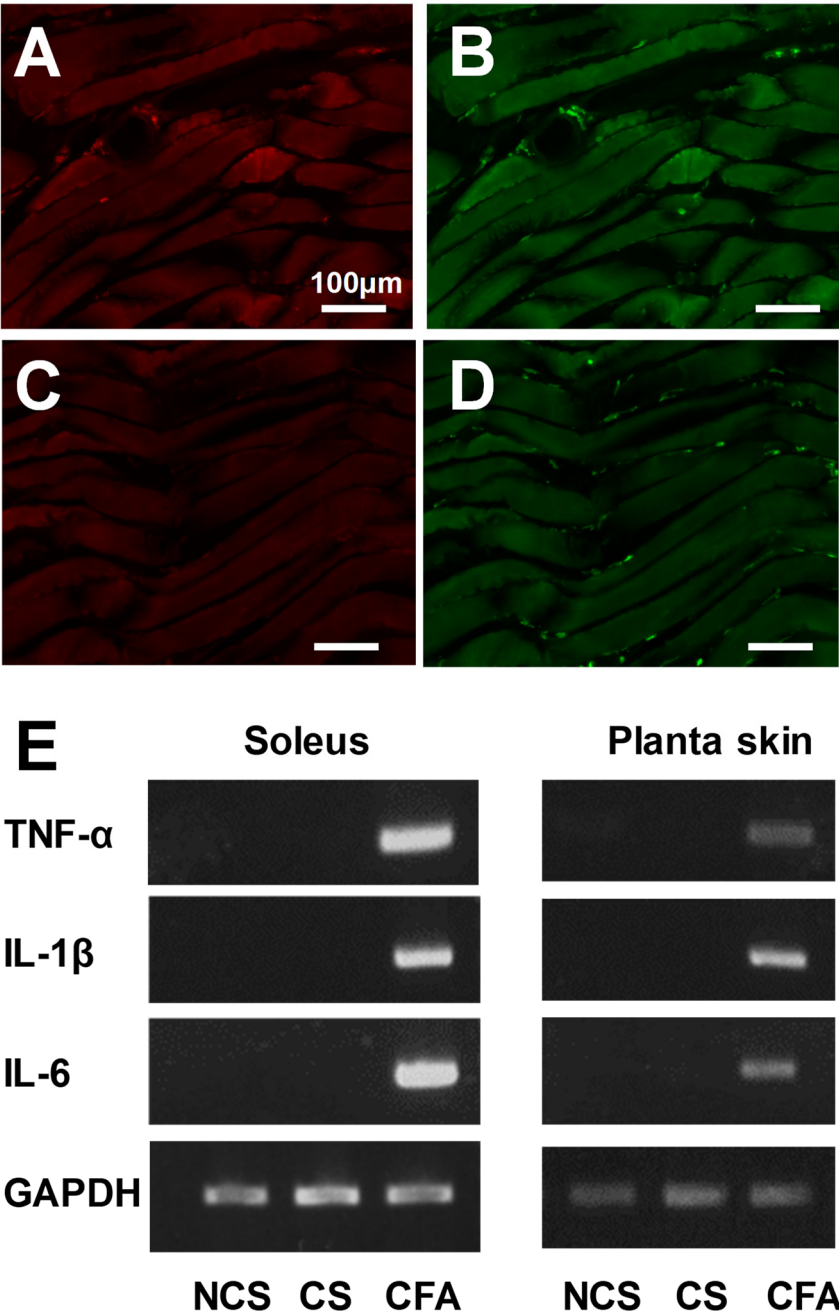

Supplement: Supplementary file 3 — Figure S2. No evidence of inflammation was observed in the soleus or planter skin. A–B: Expression of the macrophage markers OX42 (A and C) and Iba1 (B and D) was examined, although no macrophage accumulation was observed in the soleus of CS rats. Scale bar: 100 μm. E: Polymerase chain reaction (PCR) analysis was used to examine the mRNA expression of representative inflammatory cytokines. No increases in cytokine expression were observed in the CS group relative to the expression in the NCS group, although CFA injection revealed marked increases in mRNA expression in both groups (PDF 474 kb) [file 12974_2019_1456_MOESM3_ESM.pdf]
